# Amniotic Fluid and Placental Membranes as Sources of Stem Cells: Progress and Challenges

**DOI:** 10.3390/ijms23105362

**Published:** 2022-05-11

**Authors:** Tullia Maraldi, Valentina Russo

**Affiliations:** 1 Department of Biomedical, Metabolic and Neural Sciences, University of Modena and Reggio Emilia, Via del Pozzo 71, 41125 Modena, Italy; 2 Faculty of Bioscience and Agro-Food and Environmental Technology, Agriculture and Environment, University of Teramo, Via Renato Balzarini 1, 64100 Teramo, Italy

The intention of this special edition is to collect review and original research articles that illustrate and stimulate growing efforts to understand the implication of perinatal stem cells in pathological conditions such as cardiovascular and metabolic diseases and inflammatory, autoimmune, musculoskeletal, and degenerative diseases.

Significant recent advances in regenerative therapy have intensified the research on different sources of stem cells. Although embryonic and adult tissues can be used for the isolation of pluripotent stem cells, significant limitations, including ethical concerns and complexity of isolation/culture and tumorigenicity, have hindered translation of laboratory findings into clinical practice.

Stem cell research drew attention toward amniotic membrane and amniotic fluid stem cells, since these sources possess many advantages: First of all, these cells can be extracted from discarded fetal material; secondly, abundant stem cells can be obtained; finally, these stem cell sources are free from ethical considerations and tumorigenicity. Stem cells derived from amniotic fluid and membranes possess embryonic stem cell-like differentiation capabilities and, similarly to mesenchymal stem cells, they are also able to modulate the local immune response. Their reduced immunogenicity and immunomodulatory properties allow their use in allo- and xeno-transplantation settings. These, among other properties, make these cells attractive for cellular therapy.

Perinatal stem cells in general have been regarded as an attractive and available cell source for medical research and clinical trials in recent years, and mounting evidence has demonstrated the efficacy of amnion-derived stem cells therapies in vitro and in vivo: These placental cells include even amniotic epithelial stem cells and amniotic mesenchymal stem cells. Indeed, as described in the review by Qiu et al. [1], multiple stem cell types have been identified in the human placenta. The placenta is a temporary organ that forms during pregnancy and plays a pivotal role in exchanges between the fetus and maternal blood, establishing an immune-privileged environment for fetal growth and secreting hormones that are essential for gestation. Thus, it is reasonable to investigate the characteristics of these fetal cells for their potential application value in stem cell therapy and regenerative medicine. Recent advances in knowledge on placental stem cells have revealed that human amniotic epithelial stem cells (hAESCs), due to their characteristics, can be used as a novel potential cell source for cellular therapy and clinical application. hAESCs are known to possess stem-cell-like plasticity, immune-privilege, and paracrine properties. In addition, non-tumorigenicity and a lack of ethical concerns are two major advantages compared with embryonic stem cells (ESCs) and induced pluripotent stem cells (iPSCs). More importantly, clinical research has indicated that the implantation of hAESCs or amniotic tissue in patients does not trigger an immune rejection, which might indicate their potential use as a solution to the graft-rejection issue common in ESCs and iPSCs. Of note, preclinical experiments on animals have proven that hAESCs would not form tumors upon transplantation into either immunocompetent or immunodeficient mice, guaranteeing their safety for use in further clinical applications. Furthermore, since million of hAESCs can be obtained from a term placenta, there is no need to increase cell quantity by in vitro culture for single clinical application. On the other hand, with the fast development of the serum-free culture system, isolation and culturing methods for hAESCs have become normalized and convenient, making large-scale clinical use possible. Taken together, these properties make hAESCs attractive for cellular therapy. For further clinical application, more research should be directed towards identifying the most suitable gestational age, region of cell isolation on placenta, isolation protocols, cross-contamination, passage number, and epithelial-to-mesenchymal transition (EMT). Alongside the rapid development of biomaterials aiming to support and protect cells being used for therapy, the combination of hAESCs and biomaterials is a promising tissue engineering method. All characteristics mentioned above make hAESCs a potential ideal cell type for use in both research and regenerative medicine in the near future.

The review by Liu et al. [2] supports these considerations, highlighting the demonstrated advantages of human amnion-derived stem cells (hADSCs) including human amniotic mesenchymal stem cells (hAMSCs) and human amniotic epithelial stem cells (hAESCs) over other stem cells. The authors describe the biological characteristics and advantages of hADSCs, especially for their high pluripotency and immunomodulatory effects, and summarize their therapeutic applications for preclinical research. More recently, numerous clinical trials have been conducted throughout the world for evaluating the safety and efficacy of hADSCs in different diseases. Liu et al. highlight the properties of hADSCs as a promising source of stem cells for cell therapy and regenerative medicine for treatments of a wide range of diseases, even if more studies are needed to further investigate the pluripotent properties and to elucidate the mechanisms of their paracrine effects. In fact, accumulating evidence has demonstrated that hADSCs are able to activate endogenous mechanisms of tissue regeneration by secreting bioactive cytokines and exosomes that achieve a positive outcome, representing a promising alternative for stem cell-free therapies. In addition to paracrine effects, hADSCs also have strong capability to transdifferentiate, for example, into cardiomyocytes, hepatocytes, epidermal cells, corneal epithelial-like cells, neural cells, and functional insulin-secreting cells in vitro following chemical induction, biological treatment, gene transfection, or coculture with other types of cells, suggesting that, in the future, inducing the differentiation of hADSCs toward a desired phenotype in vitro before transplantation will be an effective method for several treatments.

In this context, amnion-derived stem cells have been demonstrated to transdifferentiate toward neuroectodermal cells, as reported in the study of Gaggi et al. [3]. The degeneration of dopaminergic neurons represents the cause of many neurodegenerative diseases, with increasing incidences worldwide. The replacement of dead cells with new healthy ones may represent an appealing therapeutic approach to these pathologies, but currently, only pluripotent stem cells can generate dopaminergic neurons with high efficiency. However, the use of these cells induces safety and/or ethical issues. Human mesenchymal stromal cells (hFM-MSCs) are perinatal stem cells that can be easily isolated from the amniochorionic membrane after delivery. Generally considered multipotent, their real differentiative potential is not completely elucidated. The aim of this study was to analyze their stemness characteristics and to evaluate whether they may overcome their mesenchymal fate, generating dopaminergic neurons. This study demonstrated that hFM-MSCs expressed embryonal genes OCT4, NANOG, SOX2, KLF4, OVOL1, and ESG1, suggesting they have some features of pluripotency. Moreover, hFM-MSCs that underwent a dopaminergic differentiation protocol gradually increased the transcription of dopaminergic markers LMX1b, NURR1, PITX3, and DAT. They obtained a homogeneous population of cells resembling the morphology of primary midbrain dopaminergic neurons that expressed functional dopaminergic markers TH, DAT, and Nurr1. In conclusion, these results suggested that hFM-MSCs retained the expression of pluripotency genes and are able to differentiate not only into mesodermal cells but also into neuroectodermal dopaminergic neuron-like cells.

Regarding the paracrine effect, it has been shown that the conditioned medium (CM) derived by amniotic membrane stem cells also possesses a therapeutic role. Miceli et al. [4] in their article have shown that the CM of human amnion-derived MSCs (hAMSCs) in an in vitro model of lung ischemia-reperfusion injury (IRI) can have protective effects on ischemic-injured pulmonary cells by delaying cells entering apoptosis and preserving cell viability and membrane integrity, partially through the downregulation of inflammatory factors and the upregulation of antiapoptotic factors. In particular, 3D hAMSC cultures showed the increased production of both immunosuppressive (IL-4, IL-10, IL-1RA, HGF, and LIF) and growth factors (EGF, FGF-2, HGF, and PIGF-1), inhibiting crucial inflammatory factors (NF-kB, IL-1β, IL-6, IL-8, and MCP-1) and inducing a key antiapoptotic factor (BCL2) in injured cells attenuating IRI effects. These authors conclude that the addition of hAMSC-CM to the conventional lung preservation solution could improve the efficacy of ex vivo lung perfusion, leading to strategies for a potential implementation of this technique.

In Ciardulli et al. [5] also highlighted the regenerative potential of another type of perinatal stem cell, such as the extra-embryonic Wharton’s Jelly derived mesenchymal stem cells (WJ-MSCs), as an emerging and useful alternative stem cell source, especially for tendon regeneration. In their article, the authors explored the tenogenic responsiveness of hWJ-MSCs to human Growth Differentiation Factor 5 (hGDF-5) and compared the outcome to hBM-MSCs. They have demonstrated that hGDF-5 supplementation determined in hWJ-MSCs improved tendon-related marker up-regulation with respect to hBM-MSCs. Moreover, hWJ-MSCs also showed larger proliferation rate and marked aggregation into a tubular-shaped system. Both cell types showed specific alignment and shape modification with a length/width ratio increase, suggesting their response in activating tenogenic commitment events. Simultaneous to this, Ciardulli et al. explored the expression of pro-inflammatory (IL-6, TNF, IL-12A, and IL-1β) and anti-inflammatory (IL-10 and TGF-β1) cytokines in both cell types showing similar results. Their data suggested that, given their properties, hWJ-MSCs could be potentially used in tendon tissue engineering (TE).

Indeed, in vitro techniques are fundamental to the study of tendon development, healing, and regeneration. Citeroni et al. [6], in fact, in their review highlight the importance to identify key molecular and cellular processes involved in the progression of tendinopathies to develop effective therapeutic strategies and to drive the tissue toward regeneration. TE can be considered amongst innovative strategies. TE involves the use of a combination of key factors, such as cells, scaffolds, biochemical inputs, and mechanical inputs to produce a functional tissue-like construct. Cells represent the building blocks of the engineered tissue. Several studies displayed the involvement of various types of stem cells from different sources, amongst which include amniotic membrane and amniotic fluid stem cells, with promising results. Scaffolds supply mechanical stability and provide a 3D support for cell growth and differentiation. Mechanical inputs with different loading features, provided by bioreactors, can dynamically affect the cell’s behaviour within the scaffold, mimicking the physiological environment of the tendon. On the other hand, biologically active molecules (such as growth factors) or hypoxia can be used in synergy with the other factors to drive the process of cells maturation and differentiation. Taken together, all these elements contribute to the formation of a tissue-engineered substitute to be used as an in vitro model or to be applied in tissue replacement techniques in vivo. The authors conclude that the in vitro techniques are fundamental to study tendon development, healing, and regeneration in order to translate the knowledge in vivo to treat tendon disorders.

Regarding amniotic fluid stem cells (AFSCs), the review by Fang et al. [7] deeply analyzed the literature and reported that AFSCs can expand in vitro more than 150 times without the shortening of telomerase and without being tumorigenic after transplantation in animal models. In fact, AFSCs express specific embryonic antigens, such as SSEA-4 and CD90, that are specific markers in the ESCs, but they do not express CD45, CD34, CD133, or other hematopoietic stem cell markers. In addition, more than 90% of the cells express transcription factor Oct4. These findings indicate that the differentiation ability of AFSCs lies between multipotent and pluripotent stem cells. The low immunogenicity derives from the weakly positivity for MHC class II of these cells, while they are positive for major histocompatibility (MHC) class I molecules, which are also called HLA-ABC. These advantages allow AFSCs to be an ideal candidate for use in regenerative medicine. However, the AF cell population includes several cell types from different origins. Based on different morphologies, AFSCs, collected by amniocentesis scheduled between the 15th and 18th weeks of pregnancy, can be roughly divided into two types of adherent cells—spindle-shaped (SS) and round-shaped (RS) AFSCs, accounting for approximately 6% and 94% of the total cells, respectively. SS-AFSCs show a higher ability to proliferate than RS-AFSCs. Both types of AFSCs do not express CD34, CD133, CD31, CD45, CD14, and HLA-DR. However, they express the markers of MSCs, such as CD73, CD105, and CD166, as well as adhesion molecules, such as CD29, CD44, CD49e, and HLA-ABC. The selection of AFSCs based on different subtypes can increase their efficiency for preclinical and clinical applications.

Due to the differentiation ability of AFSCs and low immunogenicity than other allogeneic-transplanted pluripotent stem cells, AFSCs are considered as potential candidates that can compensate for the limitations of pluripotent and multipotent stem cells.

This review by Fang et al. finally recapitulates how AFSCs could be particularly valuable in restoring cardiac tissue after myocardial infarction or other cardiovascular diseases. Since adult cardiomyocytes fail to regenerate, the heart loses the ability to repair itself after an injury, making patients with heart disease suffer from poor prognosis. Pluripotent stem cells have the ability to differentiate into cardiomyocytes in vitro through a well-established process, which is a new advancement in cardiac regeneration therapy. However, pluripotent stem cell-derived cardiomyocytes have certain drawbacks, such as the risk of arrhythmia and immune incompatibility. The possibility of development of cardiomyocytes from AFSCs, as well as their transplantation in host cells to produce mechanical contraction, is of certain interest. Thus, this review article highlighted the progress of AFSC therapy and its application in the treatment of heart diseases in recent years. In summary, the intramyocardial transplantation of AFSCs in infarcted rat hearts improved post-infarct cardiac function, but only a small number of the transplanted AFSCs could survive in the hearts and differentiate into endothelial cells and smooth muscle cells. Moreover, these transplanted AFSCs minimally differentiated into cardiomyocyte. Thus, apart from differentiating into cardiomyocytes, AFSCs also provide mechanical contractility to the host heart and may also repair injured hearts through paracrine signaling.

This topic was the focus of the study by Gasiuniene et al. [8]. They investigated the potential of biologically active compounds, namely, angiotensin II, retinoic acid (RA), epigallocatechin-3-gallate (EGCG), vitamin C alone, and the combinations of RA, EGCG, and vitamin C with angiotensin II to induce cardiomyogenic differentiation of AFSCs. We observed that the upregulated expression of cardiac gene markers (NKX2-5, MYH6, TNNT2, and DES) and cardiac ion channel genes (sodium, calcium, and potassium) as well as the increased levels of Connexin 43 and Nkx2.5 proteins. Extracellular flux analysis, applied for the first time on AFSCs induced with biologically active compounds, revealed the switch in AFSCs energetic phenotype and the enhanced utilization of oxidative phosphorylation for energy production. Moreover, they demonstrated changes in epigenetic marks associated with transcriptionally active (H3K4me3, H3K9ac, and H4hyperAc) or repressed (H3K27me3) chromatin. All in all, these explored biomolecules were able to induce alterations in AFSCs at the phenotypic, genetic, protein, metabolic, and epigenetic levels, leading to the formation of cardiomyocyte progenitors that may become functional heart cells in vitro or in vivo.

The capacity to regulate inflammation is typical of mesenchymal stem cells, including AFSCs. A systemic inflammatory response induces multiple organ dysfunction and results in poor long-term neurological outcomes in neonatal sepsis. However, there is no effective therapy for treating or preventing neonatal sepsis besides antibiotics and supportive care. Therefore, a novel strategy to improve neonatal sepsis-related morbidity and mortality is desirable. The group of Abe et al. [9] recently reported that prophylactic therapy with AFSCs improved survival in a rat model of lipopolysaccharide (LPS)-induced neonatal sepsis through immunomodulation. In addition to improving mortality, increasing survival without major morbidities is an important goal of neonatal intensive care for neonatal sepsis. In the study published in this Special Issue, they investigated long-term neurological outcomes in neonatal sepsis survivors treated with AFSCs using the LPS-induced neonatal sepsis model in rats. Prophylactic therapy with hAFSCs improved spatial awareness and memory-based behavior in neonatal sepsis survivors at adolescence. The treatment suppressed acute reactive gliosis and subsequently reduced astrogliosis in the hippocampal region over a long period of assessment. They demonstrated the efficacy of AFSC therapy in improving the mortality and morbidity associated with neonatal sepsis.

The therapeutic potential of transplanted stem cells has been broadly shown to be mostly mediated by their secreted soluble factors, which can orchestrate a pro-regenerative microenvironment in the host tissue while triggering the activation of endogenous mechanisms of functional recovery. In addition to pluripotent characteristics and immune privileges, AFSCs showed unique paracrine properties in the preclinical models of several diseases. AFSCs can secrete vesicles through automatic paracrine signaling or via stimulation. Vesicles secreted by AFSCs may also contain microRNA. It has been confirmed that these vesicles can reduce oxidative stress in vitro and reduce cell apoptosis through the enrichment of exosomes containing regulatory microRNA. The immune tolerance and regulation capabilities of AFSCs have been evaluated by using a peripheral blood mononuclear cell (PBMC) co-culture in vitro; AFSC inhibited the activation of B cells and reduced the inflammation in vivo. In addition, AFSCs exhibited the ability to generate vascular endothelial cells.

The study by Costa and co-workers [10] is focused on the secretome derived from stem cells of amniotic fluids obtained during amniocentesis or the time of delivery. They previously reported that c-KIT+ human amniotic-fluid derived stem cells obtained from leftover samples of routine II trimester prenatal diagnosis (foetal hAFS) are endowed with regenerative paracrine potential driving pro-survival, anti-fibrotic, and proliferative effects. However, due to the new cytogenetic analysis procedure, amniocentesis is not common any more, but hAFS may also be isolated from III trimester clinical waste samples during scheduled C-sections (perinatal hAFS), thus offering an easily accessible alternative to fetal hAFS. This study provided a detailed characterization of the hAFS total secretome (i.e., the entirety of soluble paracrine factors released by cells in the conditioned medium, hAFS-CM) and the extracellular vesicles (hAFS-EVs) within it, from II trimester fetal- versus III trimester perinatal cells. Moreover, fetal- and perinatal hAFS were characterized and subject to hypoxic preconditioning to enhance their paracrine potential. hAFS-CM and hAFS-EV formulations were analyzed for protein and chemokine/cytokine content, and the EV cargo was further investigated by RNA sequencing. The phenotype of fetal- and perinatal hAFS, along with their corresponding secretome formulations, overlapped. The profiling of their paracrine cargo revealed some differences according to the gestational stage and hypoxic preconditioning. Both cell sources provided formulations enriched with neurotrophic, immunomodulatory, anti-fibrotic, and endothelial stimulating factors, and the immature foetal hAFS secretome was defined by a more pronounced pro-vasculogenic, regenerative, pro-resolving, and anti-aging profile. Small RNA profiling showed microRNA enrichments in both fetal- and perinatal hAFS-EV cargo, with a stably expressed pro-resolving core as a reference molecular signature. Here, it was confirmed that hAFS represents an appealing source of regenerative paracrine factors; the selection of either foetal or perinatal hAFS secretome formulations for future paracrine therapy should be evaluated by also considering the specific clinical scenario.

The therapeutic efficacy of EVs derived from AFSCs was demonstrated in other chronic diseases. The study by Gatti et al. [11] evaluated the modulating role of AFSC vesicles on an in vitro model of osteoporosis. Current therapies for osteoporosis are mostly concentrated on how to inhibit bone resorption but produce serious adverse effects. Therefore, more effective and safer therapies are needed that even encourage bone formation. Human AFSC-EVs were added to the culture medium of a human pre-osteoblast cell line (HOB) induced to differentiate, and then they were treated with dexamethasone as an osteoporosis inducer. AFSC-EVs were able to ameliorate the differentiation ability of HOB both in the case of pre-osteoblasts and when the differentiation process was affected by dexamethasone. Moreover, viability increased, and in parallel, apoptotic markers were reduced. Since steroids induce oxidative stress, the levels of reactive oxygen species and of redox related proteins were evaluated. The presence of EV positively modulated redox unbalance due to dexamethasone. The authors concluded that EV from hAFSCs have the ability to recover precursor cell potential and delay local bone loss in steroid-related osteoporosis.

In conclusion, citing Costa et al., stem cell vesicles from discarded perinatal tissues have been increasingly proposed as an innovative therapy medicinal product by multiple independent preclinical studies targeting cardiovascular, neurological, and/or inflammatory disease. Accordingly, stem cells may be envisioned as biological factories for the exploitation of their therapeutic secretome by offering ready-to-use and off-the-shelf regenerative treatments.
